# Screen-based sedentary behavior and associations with functional strength in 6–15 year-old children in the United States

**DOI:** 10.1186/s12889-016-2791-9

**Published:** 2016-02-04

**Authors:** Lisa R. Edelson, Kevin C. Mathias, Victor L. Fulgoni, Leonidas G. Karagounis

**Affiliations:** 1 Nestlé Research Center, Case Postale 44, CH-1000 Lausanne, Switzerland; 2 Nutrition Impact, 9725 D Drive North, Battle Creek, MI 49014 USA

**Keywords:** Children, Sedentary behavior, Screen time, Strength, Television, NHANES

## Abstract

**Background:**

Physical strength is associated with improved health outcomes in children. Heavier children tend to have lower functional strength and mobility. Physical activity can increase children’s strength, but it is unknown how different types of electronic media use impact physical strength.

**Methods:**

Data from the NHANES National Youth Fitness Survey (NNYFS) from children ages 6–15 were analyzed in this study. Regression models were conducted to determine if screen-based sedentary behaviors (television viewing time, computer/video game time) were associated with strength measures (grip, leg extensions, modified pull-ups, plank) while controlling for potential confounders including child age, sex, BMI z-score, and days per week with 60+ minutes of physical activity. Grip strength and leg extensions divided by body weight were analyzed to provide measures of relative strength together with pull-ups and plank, which require lifting the body.

**Results:**

The results from the regression models showed the hypothesized inverse association between TV time and all strength measures. Computer time was only significantly inversely associated with the ability to do one or more pull-ups.

**Conclusions:**

This study shows that television viewing, but not computer/videogames, is inversely associated with measures of child strength while controlling for child characteristics and physical activity. These findings suggest that “screen time” may not be a unified construct with respect to strength outcomes and that further exploration of the potential benefits of reducing television time on children’s strength and related mobility is needed.

## Background

Children’s physical strength and cardiorespiratory fitness are related to a variety of health outcomes such as risk of cardiovascular, musculoskeletal, and metabolic conditions, as well as improvements in general mobility and physical functioning [1–4]. The importance of maintaining adequate levels of physical activity throughout life is widely recognized [5]. Given the plethora of research on the benefits of physical activity, current physical activity guidelines for children (>6 years of age) and adolescents in the US recommend at least 60 minutes per day of moderate- to vigorous-intensity physical activity (MVPA) and at least 3 times a week of weight bearing exercises to promote muscle and bone strength (Physical Activity Guidelines for Americans, U.S. Dept. of Health and Human Services). Similar recommendations are in place globally [6].

Inactivity is widely accepted to be related to increased BMI [7, 8], but the relationship between BMI and strength, at first glance, seems to be mixed. A study by Ervin and colleagues [9] found that obese and overweight children in the United States had greater grip- and leg strength, but lower core and upper-body strength than normal weight children. This difference in findings between the two sets of strength measures likely lies in the fact that the core and upper-body strength measures used (plank and a modified pull-up, respectively) both involve lifting the child’s own body, whereas the grip strength and leg extensions used absolute measures of strength (number of pounds of force squeezed or pushed). This distinction between absolute strength (e.g., how much weight an individual can lift at the gym) and relative or functional strength (what that person can do with their own body) is an important one, with the latter being likely more relevant to the individual’s mobility and quality of life. As overweight and obese children often present with compromised locomotor function compared to non-obese children [10], it is important to consider their strength in relation to their body size. It is for this reason that assessments in clinical settings generally adjust for the patient’s weight status when judging their ability to perform daily tasks [e.g., [11]]. In the current study, we focused on children’s relative strength by dividing all measures of absolute strength (e.g., amount of force in leg extensions) by the child’s weight (kg) to get a sense of how the strength could be used functionally by the child (e.g., to walk up stairs).

Although the role of physical activity in building strength is well documented, less is known about the relationship between sedentary behaviors such as screen time (watching television, playing video games) and children’s strength. In the past, it has been hypothesized that sedentary behavior may simply reflect a lack of physical activity, but studies have shown that these two behaviors are not mutually exclusive [12, 13]. This is particularly problematic in studies which use measures of sedentary behavior, such as television viewing time, simply as a proxy for physical activity levels [14, 15]. In the current study, we specifically explore the relationship between the amount of time spent in different screen-based sedentary behaviors and strength while controlling for reported physical activity.

The objective of the present study was to utilize the available data from the 2012 National Health and Nutrition Examination Survey (NHANES) National Youth Fitness Survey (NNYFS) to explore the relationship between different screen time activities and strength in a nationally representative sample of US youth. To this end, we report on the relationship between strength of core-, lower- and upper body muscles and screen time in children between 6–15 years of age in the US.

## Methods

The National Health and Nutrition Examination Survey (NHANES) is a series of nationally representative surveys of the health and nutritional status of adults and children in the United States, comprising both questionnaire and physical examination components. The NHANES National Youth Fitness Survey (NNYFS) was conducted in 2012 as a supplement to the main NHANES survey. It was designed to collect data on physical activity and fitness in American children ages 3 to 15 years [16]. Fitness tests were administered to learn more about the physical health of the children in this age group.

### Participants

The sampling for the 2012 NNYFS [17] was conducted in conjunction with NHANES. Both samples were selected using a multistage, stratified area probability samples of non-institutionalized individuals and were weighted to provide nationally representative estimates for the United States population. Of the households selected to participate in the NNYFS, 98.5 % responded to the screening invitation. The interview response rate was 79.4 % and the examination response rate was 96.1 % (conditional on completing the interview). Additional details on the study design and measures have been published by Johnson and colleagues [16]. Not all of the strength measures were conducted with children less than 6 years of age, therefore the current analyses were restricted to children aged 6–15 years. The final sample included 1224 children.

### Procedures

Demographic, socioeconomic, physical activity, and electronic media use (e.g., television viewing) were recorded during a household interview [17]. Information from 6–11 year-old children was obtained from proxy respondents and 12–15 year-old children answered the physical activity and media use questions themselves. Anthropometric measurements (height and weight) and strength measures were obtained by trained examiners in a mobile examination center [18].

#### Physical activity

One questionnaire item assessed physical activity. Physical activity was reported as the number of days in the past week that a child spent a total of at least 60 minutes participating in any physical activity that increased heart rate or resulted in breathing hard some of the time [19]. The response values ranged from 0 to 7 days.

#### Electronic media use

Two separate questions provided information about children’s electronic media use. Television time was reported as the average number of hours per day over the past 30 days that a child spent watching television or videos. Computer usage was reported as the average number of hours per day over the past 30 days that a child used a computer or played video games outside of work- or school-related activities, not including time previously mentioned in the TV item (if children watched videos on the computer) [19]. For each question, participants could respond that they did not do this activity (0 hours), or that they did it on average for less than an hour, 1 hour, 2 hours, 3 hours, 4 hours, or 5 or more hours per day.

#### Strength

Four different measures of physical strength were measured in the mobile examination center: grip strength, leg extension strength, number of modified pull-ups and plank time. Grip strength assessed upper-body muscle strength using a hand-grip dynamometer (Takei Digital Grip Strength Dynamometer, Takei Scientific Instruments Co., Ltd). Participants were instructed to squeeze the handle of the dynamometer as hard as possible three times with each hand [20]. The strongest of the six measurements was included in the analysis. Leg extension strength assessed lower-body strength using a belt-stabilized hand-held dynamometer (HHD, MicroFET2, Hoggan Health Industries). Participants sat in a chair and were restrained at the hips, thighs and trunk with web belts. The dynamometer was placed perpendicular to the shin near the ankle bones and the participants were instructed to press their leg forward as hard as possible into the dynamometer [21]. Three measurements were taken for each leg and the strongest of the six measurements was included in the analysis. The plank test (a horizontal body position held with only the hands/forearms and toes touching the floor) assessed core muscle strength and endurance. Participants started lying face down on a mat and then the duration (in seconds) that they could hold the plank position was measured [22]. The modified pull-up assessed upper-body strength. Participants lay on their backs and used their arms to pull themselves up to a strap hanging down 8 inches from the center of the bar [23]. The number of completed pull-ups was included in the analysis. It should be noted that both the grip and leg strength tests assessed absolute muscle strength, whereas the modified pull-up and plank tests involve lifting the weight of the body and therefore are relative measures of strength/endurance in proportion to body weight. In addition to analyzing absolute grip and leg strength, these measures were also converted into relative strength measures with respect to body weight by dividing the absolute grip and leg strength recorded by the weight of the child (kg).

### Data analysis

For the descriptive analyses of physical activity, TV and computer time, and absolute- and relative strength measures, children’s data are presented divided into three age groups that roughly represent elementary-school age (6–9), pre-adolescence (10–12), and early adolescence (13–15). Initially, simple linear regression models were used to examine trends across these variables. Due to the potential of rounding and the discrete nature of TV and computer time, a non-parametric test extending the Wilcoxon rank-sum test was conducted to test for trends across the ordered age groups to confirm the robustness of the findings from the simple linear regression models.

Additionally, a series of multiple linear regression models controlling for age, sex, physical activity, and BMI *z*-score were utilized to determine the associations of television time and computer time with absolute- and relative strength measures. The number of hours of both television time and computer time were modeled as continuous variables. Children reporting “<1 hour” were assigned the midpoint of 0.5 hours given that it was also possible to report 0 or 1 hour. The questionnaire limited the maximum number of hours children were able to report to a maximum of “5 hours or more” of TV or computer time per day. Children in this category were assigned a value of 5 hours in the regression models.

Due to the high percentage of children who were unable to complete one modified-pull, the appropriateness of an ordinary least-squares regression model was questionable for that portion of the analysis. For the adjusted analysis of modified pull-ups, both a logistic (0 versus ≥1 modified pull-up) and a linear regression model conditional on the child being able to complete at least 1 modified pull-up were conducted to address the analytical issue of high percentages of zeroes in the data. The logistic regression model estimated the change in the odds that a child could complete at least one modified pull-up and the regression model estimated the change in number of modified pull-ups completed, using only children that could complete at least one modified pull-up. For all models, both television and computer time were included simultaneously to examine if there were separate pathways by which television and computer time were associated with strength.

The statistical tests for trends across the three age categories were conducted using STATA 13.1 (StataCorp, College Station, TX). All other analyses were conducted using SAS 9.3 (SAS Institute Inc.; Cary, NC) and SUDAAN 11 (Research Triangle Institute, Research Triangle Park, NC, USA). With the exception of the sensitivity analysis utilizing an extension of the Wilcoxon rank-sum non-parametric test (*nptrend* command, STATA 13.1), all analyses accounted for the complex survey design of the NHANES datasets. Analytical weights based on probabilities of selection and participation in the study were utilized, resulting in estimates representative of the US population. Primary sampling units (geographical area) and strata from the first stage of the sampling design were accounted for in estimation of the standard errors. A criterion of (*p* < 0.05) was used for all tests of statistical significance.

### Ethics, consent, and permissions

The National Center for Health Statistics (NCHS) Research Ethics Review Board approved all NHANES protocols. A parent or legal guardian of each participant provided informed consent and participants who were at least 7 years old also provided written assent before any questionnaires or measurements were administered.

## Results

Characteristics of the analytical sample are provided in Table 1. Of the original 1224 participants, 26 subjects were missing data for grip strength, 34 were missing leg strength, 29 were missing modified pull-up data, and 21 were missing plank data.

**Table 1:** Sample characteristics and days per week of physical activity among the 2012 NHANES National Youth Fitness Survey participants

	6-9 y	10-12 y	13-15 y	Full sample (6–15 y)
	*n* = 491	*n* = 379	*n* = 354	*n* = 1224
Subject Charateristics	% of sample^b^	% of sample^b^	% of sample^b^	% of sample^b^
Sex				
Female	47	50	50	49
Race/Ethnicity				
non-Hispanic White	52	54	55	54
non-Hispanic Black	14	12	15	14
Hispanic	22	24	22	23
Other	11	10	8	10
Weight Status^c^				
Underweight	3	4	3	3
Normal Weight	65	53	61	60
Overweight	14	25	17	18
Obese	19	19	20	19
Days of Physical Activity per Week^a^				
0 days	2	5	7	4
1-3 days	10	19	29	18
4-6 days	26	32	40	32
7 days	62	43	24	46
Mean (95 % CI) (Days)^d^	5.9 (5.7 – 6.1)***	5.1 (4.8 – 5.3)	4.4 (4.2 – 4.6)	5.2 (5.0 – 5.4)

^a^Physical activity was self- or proxy reported as the number of days that the child was physically active (increased heart rate or breathing hard some of the time) for a total of at least 60 minutes in the past 7 days)

^b^Nationally representative estimates taking into account the survey design of the 2012 NNYFS

^c^Weight status of children were based on BMI-for-age percentiles

^d^A linear trend across the three age categories for average days of physical activity was tested using a simple linear regression model accounting for the survey design of the 2012 NNYFS

***Indicates a significant regression coefficient for a linear trend (*p* < 0.001) across the three sub age groups

### Changes in activity and strength with age

Distributions of physical activity for all three age categories are reported in Table 1. A significant trend across age categories (*p* < 0.001) was shown with decreasing days spent exercising among older children. Distributions of time spent watching TV or using the computer are reported in Table 2. The results show older children spent more time watching TV and using computers/video games outside of school than did younger children (trend across age categories for TV: *p* < 0.001 and computer: *p* < 0.001). Distributions of absolute and relative strength measures are presented in Table 3. Significant increases in absolute and relative measures of children’s strength were shown with increasing age; however, older children were less likely to be able to complete at least one modified pull-up (*p* = 0.029).

**Table 2:** Number of hours per day children reported watching TV watching or playing computer/ video games

	0 Hours	<1 Hour	1 Hour	2 Hours	3 Hours	4 Hours	5+ Hours	Mean (95 % CI)^a^ (Minutes)
TV Time^b^	% of Sample							
6-9 y	1	17	30	32	12	4	3	103 (91 – 115)***
10-12 y	1	14	21	34	15	6	8	125 (111 – 138)
13-15 y	2	12	21	29	21	9	8	130 (121 – 139)
Full sample (6–15 y)	1	15	25	32	16	6	6	117 (109 – 126)
Computer Time^c^								
6-9 y	12	41	26	12	5	2	2	61 (54 – 67)***
10-12 y	12	31	24	17	10	3	3	78 (67 – 88)
13-15 y	10	23	16	20	14	10	8	114 (99 – 125)
Full sample (6–15 y)	11	33	23	16	9	5	4	80 (73 – 88)

^a^Linear trends across the three age categories for hours of TV or computer time were tested using simple linear regression models accounting for the survey design of the 2012 NNYFS. Due to the potential of rounding and discrete nature of these variables, a non-parametric test extending the Wilcoxon rank-sum test was conducted using STATA (version 13.1, 2013, StataCorp, College Station, TX) to test for trends across the ordered age groups to confirm the robustness of the findings from the linear regression models

^b^Television time estimated based on self-report of the average hours per day over the last 30 days

^c^Computer or video game time outside of work or school estimated based on self-report of the average hours per day over the last 30 days

***Indicates a significant linear trend (*p* < 0.001) across age groups for both the linear regression model (regression coefficient) and the extension of the Wilcoxon-rank sum (non-parametric) test

**Table 3:** Distributions of absolute- and relative strength measures

	Percentiles					Mean ± SE		Linear trend for difference across age groups (*p*-value)^a^	
	10	25	50	75	90				
Absolute Strength									
Grip (kg)^b^									
Age Group									
6-9 y	10	12	14	16	19	14.1 (13.7 –14.6)		<0.001	
10-12 y	16	19	22	26	31	23.1 (22.2 – 23.9)			
13-15 y	24	27	31	37	44	32.6 (31.3 – 33.8)			
Full sample (6–15 y)	11	14	20	28	36	22.2 (21.5 – 22.9)			
Leg Extensions (kg)^c^									
6-9 y	8	12	17	22	27	17.7 (14.9 – 20.5)			
10-12 y	12	22	30	36	44	29.1 (25.0 – 33.2)		<0.001	
13-15 y	18	29	41	49	59	40.2 (33.6 – 46.8)			
Full sample (6–15 y)	11	16	24	37	48	27.7 (23.4 – 32.0)			
Relative Strength									
Grip (kg per kg of Body Weight)^d^									
6-9 y	0.37	0.42	0.48	0.55	0.61	0.49 (0.47 – 0.50)			
10-12 y	0.34	0.43	0.50	0.57	0.62	0.50 (0.49 – 0.51)		0.005	
13-15 y	0.37	0.45	0.53	0.60	0.69	0.53 (0.51 – 0.55)			
Full sample (6–15 y)	0.37	0.43	0.50	0.57	0.64	0.50 (0.50 – 0.51)			
Leg Extensions (kg per kg of Body Weight)^e^									
6-9 y	0.31	0.46	0.63	0.74	0.87	0.60 (0.51 – 0.70)			
10-12 y	0.31	0.47	0.66	0.77	0.86	0.62 (0.54 – 0.70)		0.020	
13-15 y	0.30	0.47	0.65	0.82	0.94	0.65 (0.54 – 0.75)			
Full sample (6–15 y)	0.31	0.46	0.64	0.77	0.89	0.62 (0.53 – 0.71)			
Plank (Seconds)^f^									
6-9 y	15	28	51	72	104	56.9 (52.5 – 61.3)			
10-12 y	19	38	64	92	127	69.7 (64.7 – 74.7)		<0.001	
13-15 y	29	55	79	121	149	88.6 (80.3 – 96.9)			
Full sample (6–15 y)	18	37	63	91	128	69.9 (66.1 – 73.6)			
Pullups (Repetitions)^g^						% ≥ 1 (95 % CI)^h^	Mean (95 % CI)^h^		
6-9 y	0	0.5	2.9	6.5	10.2	81 (77 – 86)	5.7 (5.3 – 6.0)	0.029	<0.001
10-12 y	0	0.6	3.5	7.6	11.9	80 (76 – 85)	6.7 (5.5 – 7.9)		
13-15 y	0	0	4.7	10.1	19.5	74 (69 – 80)	9.6 (8.6 – 10.6)		
Full sample (6–15 y)	0	0.4	3.4	8.0	13.0	79 (75 – 82)	7.0 (6.5 – 7.6)		

^a^ Linear trends across the three age categories for absolute- and relative strength measures were tested using simple linear regression models accounting for the survey design of the 2012 NNYFS

^b^ Muscle strength was measured through a grip test using a handgrip dynamometer

^c^ A digital hand-held dynamometer was used to measure the knee extension force

^d^ Relative grip strength was estimated as a child’s grip strength divided by their body weight

^e^ Relative leg extension strength was estimated as a child’s leg strength divided by their body weight

^f^ A horizontal body position held with only the hands/forearms and toes touching the floor

^g^ Participants lay on their backs and used their arms to pull themselves up to a strap hanging down 8 inches from the center of the bar

^h^ Due to the large proportion of children that could not complete a modified pull-up, the analysis was conducted using two models: a logistic regression model to test a linear trend in the odds of being able to complete ≥1 modified pull-up with age, and a linear regression model to test a linear trend in the number of pull-ups with age among children that could complete 1 or more modified pull-ups

### Electronic media use and associations with strength

The results from the regression models showed the hypothesized inverse association between TV time and all strength measures (Table 4). The associations between computer time and strength measures only reached significance for the odds of completing one or more pull-ups.

**Table 4:** Associations between independent variables (TV, computer/game time, age, sex, physical activity, and BMI) and strength measures

	TV change in strength per 1 hr increase	Computer change in strength per 1 hr increase	Age change in strength per 1 year increase	Sex Males - Females	Physical Activity change in strength per 1 day increase	BMI-Z-Score change in strength per 1 z-score increase
	Beta (95 % CI)	Beta (95 % CI)	Beta (95 % CI)	Beta (95 % CI)	Beta (95 % CI)	Beta (95 % CI)
			Absolute strength			
Grip^a^ (kg)	−0.31* (−0.56, −0.06)	0.05 (−0.12, 0.22)	2.79*** (2.69, 2.89)	3.16*** (2.53, 3.79)	0.02 (−0.11, 0.15)	1.58*** (1.30, 1.86)
Leg Extensions^a^ (kg)	−0.99** (−1.70, −0.28)	0.35 (−0.42, 1.13)	3.31*** (2.75, 3.86)	1.35* (0.091, 2.62)	−0.14 (−0.57, 0.29)	3.22*** (2.46, 3.98)
			Relative strength			
Grip^a^ (kg per kg of body weight)	−0.006* (−0.011, −0.001)	−0.002 (−0.006, 0.002)	0.009*** (0.007, 0.011)	0.049*** (0.039, 0.060)	0.003** (0.001, 0.005)	−0.058*** (−0.064, −0.053)
Leg Extensions^a^ (kg per kg of body weight)	−0.018* (−0.033, −0.004)	0.001 (−0.013, 0.015)	0.009** (0.004, 0.014)	0.001 (−0.021, 0.024)	0.001 (−0.007, 0.010)	−0.048*** (−0.064, −0.033)
Plank^a^ (seconds)	−3.49*** (−5.18, −1.18)	−2.04 (−4.22, 0.13)	6.19*** (5.00, 7.39)	7.45 (−0.10, 15.01)	1.76** (0.67, 2.86)	−12.39*** (−14.53, −10.26)
Pull-ups^bc^ (odds ratio for completing ≥1 pull-ups)	0.83*** (0.76, 0.92)	0.86** (0.78, 0.94)	0.99 (0.94, 1.05)	3.41*** (2.36, 4.93)	1.05 (0.99, 1.12)	0.40*** (0.31, 0.51)
Pull-ups^b^ (# of pull-ups for children with ≥1 pull-ups)	−0.34 (−0.84, 0.15)	−0.23 (−0.61, 0.14)	0.72*** (0.53, 0.90)	2.63*** (1.77, 3.49)	0.31** (0.12, 0.49)	−1.18*** (−1.48, −0.88)

^a^ Coefficients were estimated from linear regression models including all covariates in the table

^b^ Due to the large number of children unable to complete one modified pull-up, two types of models were used to analyze the data. The first model estimated the association of the covariates with the ability of a child to complete at least one modified pull-up using a logistic regression model. The second model estimated the association of the covariates with the number of modified pull-ups completed only among children that completed at least one modified pull-up using a linear regression model. Both types of models included all covariates listed

^c^ Females were coded as the reference group (denominator) for the logistic regression model

*p < 0.05, **p < 0.01, ***p < 0.001

## Discussion

It has been previously reported that children in higher BMI categories have greater absolute measures of grip strength and leg extension power, but have lower core and upper body strength when compared to children with lower BMI [9]. However, despite greater absolute leg strength, overweight and obese children show more limited mobility compared to non-obese children [10]. For this reason, it is important to consider an individual’s strength relative to their size to better understand the efficiency with which they can move their body (e.g., walk up stairs, jump) as these are measures that can influence a child’s current and future quality of life, whereas the child’s absolute strength (e.g., lifting weights) may be a less meaningful measure of his/her physical functioning. To this end, in the current study, we reported the effect of different types of electronic media use on measures of both absolute- and relative strength in children.

The amount of time spent viewing television was significantly inversely associated with both absolute and relative strength measures, even when controlling for potentially confounding variables such as age, sex, BMI, and physical activity. These findings are consistent with previous studies that showed inverse associations between sedentary behavior and physical fitness (e.g., running a mile) or flexibility [24–26]. However, in contrast to the current findings, the one previous study (published in 1998) that explored children’s strength as an outcome found no association between television viewing and strength [25]. The differences between the two studies could be due to changes in patterns of sedentary habits and availability of screen-based media in children’s homes in the last 20 years [27–29].

It should be noted that the partial *r* ^2^ values (data not shown) showed that although television viewing time was significantly associated with child strength, this variable explains a relatively small portion of the variance in strength (~1 %), compared to other variables, such as BMI z-score (partial *r* ^2^ between 6-36 %) or child age (1-12 %). Unlike some of the child characteristics such as age or sex that cannot be changed, television viewing is a behavior that can be modified and would likely have health benefits beyond improving child strength. While television viewing alone explains a small portion of the variance in relative strength directly, it may have a larger impact on relative strength through changes in BMI. Previous intervention studies in children have shown that reducing television viewing can decrease BMI [30, 31], and a lower BMI z-score was associated with higher relative strength in the current study. These findings suggest that less television viewing, through reducing BMI, could result in a larger impact on relative strength, although this has yet to be explored experimentally.

In contrast with television viewing, which was inversely associated with several strength measures, time spent using the computer for leisure activities or playing video games was only associated with a child’s ability to complete at least one pull-up. These findings suggest that not all sedentary behaviors, or even all types of electronic media use, are equal with respect to strength outcomes, as evidenced by the stronger inverse association between TV watching and strength measures than that between computer/video games and strength. We propose that the term “screen time” should not be considered a unified construct in the literature or in intervention studies. In fact, different types of sedentary behaviors have been associated with different risks of overweight in large-scale surveys in the United States and the Netherlands [32, 33] (but not Portuguese children [34]), as well as with different levels of energy intake in experimental studies [35]. As most of the literature on sedentary behavior or “screen time” to date has focused on time spent viewing television [36, 37], it is important to consider that other types of sedentary behavior (e.g., computer time, reading) may not have the same impacts on health and should be considered separately.

Some potential explanations for why television viewing resulted in different associations with strength compared to computer-based screen time include differences in posture or tension between passive television viewing and more engaging activities. For instance, children playing computer or video games may move more or get up more frequently. It has been reported previously in young adults that playing Ms. PacMan resulted in physiological responses and energy expenditure similar to those observed during mild-intensity exercise [38], and similar responses have also been reported for children [39]. However, it should be noted that such changes are not strong enough to replace traditional physical activity for children [39]. In contrast to the physiological response reported for computer game participation, it has previously been shown that the resting metabolic rate is lower during TV viewing compared to rest, particularly in obese children [40]. More research is needed to better understand children’s behavioral and physiological processes during these different types of electronic media use, as well as during non-screen-based sedentary behaviors such as reading or doing homework.

One of the main limitations in the current study is that the measurement of time spent in screen-based sedentary behaviors and physical activity were self-reported, which could result in biased measurements of these activities. An assumption for these results is that potential miss-reporting is not systematically associated with strength (e.g., stronger children are not more likely to under-report sedentary time).

Some further limitations exist regarding the measure of computer time and video game use. The question used in the NHANES interview excluded school-related computer use, meaning that the reported computer/video game time likely underestimates the total amount of time children spent in front of a computer. Further, it is also possible that estimating the time spent on these activities is less accurate than for television viewing which is conveniently packaged into half-hour segments by programs. For these reasons, we cannot conclude absolutely that computer use does not also have a negative effect on children’s strength, but rather that further research is needed to investigate this with more sensitive measures. An additional limitation was that it was not possible to examine non-school computer use and time spent playing video games separately. Future studies examining whether gaming, other types of computer use (e.g., surfing the web), and other sedentary behaviors such as doing homework or reading have comparable effects on strength are needed.

Lastly, numerous covariates were included in the regression models to minimize confounding; however, it is possible that some of the results are due to unmeasured variables. We considered the possibility that socioeconomic status (SES) could explain some of the relationship between television viewing and strength, but when it was included in the regression models (using poverty income ratio, with 1.85 times the poverty level as a cutoff point between the lower and higher SES groups), SES was not a significant predictor of any of the strength measures. Another possibility that we were unable to control for was that television viewing could be a proxy for a generally unhealthy lifestyle which resulted in lower strength. It is also possible that children that watched more TV had less time to do muscle strengthening exercises. Future research is needed to understand if the act alone of watching TV decreases strength or if other mechanisms explain the inverse associations observed in this study. Finally, due to the cross-sectional nature of the study, we cannot rule out the possibility of reverse causality (e.g., children with weaker muscle strength could choose to spend more time watching television). Longitudinal studies would provide more insight into the directionality of this relationship.

The strengths of the current study include the use of objective tests of physical strength as outcome measures, as well as examining both relative and absolute strength. Further, the current analyses controlled for the reported number of days per week with at least 60 minutes of physical activity, in order to specifically investigate the role of screen-based sedentary behaviors themselves, rather than simply as a replacement for physical activity. Future studies would benefit, however, from more precise, objective measures of physical activity levels and sedentary time.

Other promising research directions could include a clinical trial to reduce time spent viewing television and encouraging children to engage in more play to explore the impact not only on BMI outcomes, as has been done traditionally, but also on children’s physical functioning, including functional strength. It is important to bear in mind that children’s electronic media use is evolving and more studies are needed to explore how the use of both “traditional” forms of electronic entertainment, such as television, and newer devices such as tablets and smartphones are associated with children’s body composition, energy expenditure, and strength.

The American Academy of Pediatrics guidelines are moving away from their strict recommendations that children spend no more than 2 hours per day using screens, toward suggestions of how parents can monitor and guide children’s screen-based activities, but they still emphasize the need to set limits on children’s use of electronic media. Even excluding school-related computer use, the average child in the current study spent over 3 hours per day watching television and playing on the computer. From a public health perspective, there is certainly a need to help families reduce screen time globally, and there is a growing literature suggesting that television and other passive viewing may be the key behavior to target in interventions. Reducing television time could potentially have benefits not only on children’s physical strength, but also on other health outcomes such as weight status, sleep duration and quality, cardiovascular disease, and risk of diabetes [40-42]. The current recommendations for adults to break up every hour of inactivity time with 10 minutes of standing or movement could also be applied and encouraged in children, beyond the recommended 60 minutes per day of moderate to vigorous physical activity. Studies are beginning to show that breaks in sedentary time in children are associated with better child BMI and cardiometabolic risk [43], but it remains to be seen how this might also influence children’s strength, or whether this can be successfully manipulated by intervention [44].

## Conclusions

Television viewing time explained a small portion of the variance in strength, beyond what can be ascribed to the child’s age, sex, BMI, or physical activity. However, unlike fixed factors such as age or sex, television viewing is a behavior that can be modified. Previous intervention studies have been effective in reducing screen time and, in turn, improving BMI [38, 39], but the effect on child strength has yet to be explored. Some strategies for reducing screen-based activity are to remove televisions and other devices from the bedroom [45] and to monitor and guide children’s viewing [46]. Reducing electronic media use, particularly television viewing, could impact not only child strength, but also their fitness, BMI, and other aspects of health.

## Abbreviations

BMI: Body mass index
NHANES: National health and nutrition examination survey
NNYFS: NHANES national youth fitness survey
TV: Television
